# Development of conventional PCR and real‐time PCR assays to discriminate the origins of Chinese pepper oil and herbal materials from *Zanthoxylum*

**DOI:** 10.1002/jsfa.9458

**Published:** 2018-12-13

**Authors:** Wook Jin Kim, Sungyu Yang, Goya Choi, Inkyu Park, Pureum Noh, Chang‐Seob Seo, Byeong Cheol Moon

**Affiliations:** ^1^ Herbal Medicine Research Division Korea Institute of Oriental Medicine Daejeon Republic of Korea

**Keywords:** edible oil, SCAR marker, genetic discrimination, Zanthoxyli Pericarpium

## Abstract

**BACKGROUND:**

To ensure the safety, quality and therapeutic efficacy of processed foods and herbal medicines, it is important to identify and discriminate economically motivated adulterants. *Zanthoxylum schinifolium* is sold at a higher price than other *Zanthoxylum* species and is frequently adulterated with closely related *Zanthoxylum* species because of its high demand as a Korean food ingredient and medicinal material in markets. In addition, the pericarps of three *Zanthoxylum* species (*Z. schinifolium*, *Z. bungeanum* and *Z. piperitum*) are defined as herbal medicine Zanthoxyli Pericarpium in Korean pharmacopoeias, but not *Z. piperitum* in Chinese pharmacopoeias. Further confusion arises in the morphological similarity between *Z. armatum* (adulterant) and *Z. bungeanum*. Therefore, the aim of this study was to develop a sequence characterized amplified region (SCAR) marker for discrimination of four *Zanthoxylum* species.

**RESULTS:**

With the goal of developing rapid and reliable tools for genetic discrimination of authentic Zanthoxyli Pericarpium, we designed species‐specific SCAR markers, based on ITS2 sequences, that generate amplicons of less than 200 bp. Using these markers, we established both conventional and real‐time PCR assay methods capable of differentiating samples at the species level. We validated the ability of SCAR markers to authenticate edible oil and herbal medicine, and confirmed that some herbal medicines contaminated with *Z. armatum* are being distributed as Zanthoxyli Pericarpium in Korean and Chinese markets.

**CONCLUSIONS:**

The SCAR markers and PCR methods described represent powerful tools for protecting against adulteration and ensuring standardization of processed foods and herbal medicine. © 2018 The Authors. *Journal of the Science of Food and Agriculture* published by John Wiley & Sons Ltd on behalf of Society of Chemical Industry.

## INTRODUCTION

Adulteration or contamination of food and herbal medicines with inauthentic materials affects safety, price and taste, as well as pharmaceutical properties and nutrient quality. Accordingly, certification of authenticity is very important in both contexts.^1^, ^2^ Adulteration with substitutes occurs for a variety of reasons, including price, supply shortages and homonymous nomenclature. Several analytical techniques, including gas chromatography coupled with mass spectrometry, nuclear magnetic resonance and Fourier transform infrared spectroscopy, are used to detect adulterated species or the adulteration rate.^3^, ^4^ However, some of these methods require highly trained analysts, expensive instrumentation and long periods of time.^3^ Therefore, molecular marker studies are currently being undertaken with the goal of developing advanced techniques for the detection of adulteration in processed foods and herbal medicines.^2^, ^5^

Molecular markers at the DNA level offer cheap, rapid and accurate analysis.^6^, ^7^ DNA barcoding, which distinguishes between allied botanical species, has emerged as a promising analytical method for certification of authenticity and discrimination of adulterants.^8^, ^9^ Besides *mat*K and *rbc*L, recommended by the Plant Working Group of the Consortium for the Barcode of Life (CBOL), DNA barcoding of regions such as ITS (or alternatively ITS2), *psb*A‐*trn*H, *trn*L‐F and *rps*16 has been used to identify species in plant taxa.^9^, ^10^, ^11^ In contrast to plant DNA barcode regions located in the chloroplast genome, the ITS (or ITS2) locus is common to all eukaryotic genomes, and is present in the nucleus as tandem repeats.^12^ Although botanical taxonomists recommend analysis of DNA barcodes for accurate species identification, such assays are cumbersome and time‐consuming, involving DNA amplification, electrophoresis, gel rescue and sequence analysis.^13^, ^14^, ^15^ In recent years, sequence characterized amplified region (SCAR) markers based on species‐specific sequences obtained from DNA barcodes have been used to discriminate species in raw food materials and herbal medicines.^16^, ^17^

Raw food materials are altered by various manufacturing techniques involving physical, chemical or biochemical treatments.^6^, ^7^, ^18^ Therefore, DNA fragmentation (to sizes less than 200 bp) frequently occurs in processed food such as pressed oils, cooked foods and ground material, making it difficult to detect DNA.^19^ DNA fragmentation caused by physical force, heat, pH change or enzymatic activity decreases amplification efficiency because it is difficult for primers to bind highly fragmented DNA in the annealing step of PCR.^20^ Consistent with this, some quantitative assays yield different results from raw materials and processed foods.^10^, ^20^ Therefore, accurate species discrimination is necessary to confirm the quality of processed food, and to this end it is necessary to develop analytic methods with high sensitivity. A recent study reported that the degree of DNA fragmentation affects the amplification efficiency of real‐time PCR.^20^ Real‐time PCR assays have a very low limit of detection (LOD), and it is possible to discriminate species even from fragmented DNA.^20^ In addition, SYBR Green‐based real‐time PCR assays are less costly than probe‐based assays.^21^

The genus *Zanthoxylum* (Rutaceae) consists of approximately 250 species worldwide, of which six are distributed in Korea.^22^ Among these species, three *Zanthoxylum* species, *Z. schinifolium*, *Z. piperitum* and *Z. armatum*, are distributed throughout the Korean Peninsula and traditionally have been used for food and medicinal materials. However, *Z. coreanum*, *Z. fauriei* and *Z. ailanthoides* have not been used for this purpose because these species are distributed at restricted regions such as Jeju Island or the southern seaside part of the Korean Peninsula. Especially, *Z. schinifolium* (Chinese pepper) has been used as a spice for a long time in Korea, as well as in other Asian countries.^22^, ^23^ This plant is also used to make edible oil, called Chinese pepper oil in Korea, which has a more spicy taste and distinctive flavor than other *Zanthoxylum* species, and is also used in folk remedies to treat stomachache and asthma. On the other hand, *Z. bungeanum* (Sichuan pepper) is not distributed in Korea, but is an indispensable spice and herbal medicine in China.^24^ The fruit of the Chinese pepper provides many health benefits, including various phytochemical nutrients such as polyunsaturated fatty acids, coumarins, flavonoids and alkaloids.^23^ For these reasons, Chinese pepper oil is ten times more expensive than other vegetable oils such as sesame or perilla oil.

Some *Zanthoxylum* species are used as herbal medicines as well as food materials. Zanthoxyli Pericarpium, the name of a herbal medicine, is prescribed to treat abdominal pain, toothache, dyspepsia, vomiting, diarrhea, ascariasis and eczema in traditional Korean and Chinese medicine, but these national pharmacopoeias differ in how they define botanical species.^23^, ^24^, ^25^ In the Korean pharmacopoeia, the pericarps of *Z. schinifolium*, *Z. piperitum* or *Z. bungeanum* are defined as the authentic herbal medicine Zanthoxyli Pericarpium, whereas the Chinese pharmacopoeia describes only two species, *Z. schinifolium* or *Z. bungeanum*.^25^ Due to insufficient production in Korea, large amounts of Zanthoxyli Pericarpium are supplied from China. In addition, the morphological characteristics of *Z. armatum*, an inauthentic adulterant species, are very similar to those of authentic *Z. bungeanum*. Therefore, the identification of authentic material is necessary for quality control and prevention of adulteration.

In this study, we analyzed the DNA barcode sequences using ITS2 universal primers for four *Zanthoxylum* species. Candidate SCAR markers, designed using species‐specific sequences from 22 samples, were validated for the establishment of conventional and real‐time PCR assays using SYBR Green. We validated the species discrimination ability of these approaches using the SCAR markers we developed to authenticate edible oil and commercial herbal medicines.

## MATERIALS AND METHODS

### Materials

Twenty‐two samples from four *Zanthoxylum* species utilized as food ingredients or herbal medicines, *Z. schinifolium*, *Z. piperitum*, *Z. armatum* and *Z. bungeanum*, were subjected to analysis of DNA barcode sequences and used to establish PCR assay methods (Table 1). All samples were collected from native habitats or herbal medicines in Korea and China, and were identified based on botanical features by the Classification and Identification Committee of the Korea Institute of Oriental Medicine (KIOM), which consists of experts in the fields of plant taxonomy, botany, ecology, pharmacognosy and herbology. The collected materials were preserved as voucher specimens in the KIOM herbarium (IH, index herbariorum code: KIOM). Fresh leaves without lesions were stored at −75 °C prior to genomic DNA extraction.

**Table 1:** List of plant materials analyzed in this study

Number	Scientific name	Collection site	Voucher number	NCBI accession	Abbreviation
1	*Zanthoxylum schinifolium*	Seongsan, Gangneung, Gangwon‐do	KIOM2012KR11‐21	MH321526	ZS_GN
2		Gyegok, Haenam, Jeollanam‐do	KIOM2012KR14‐13	MH321527	ZS_HN
3		Danwol, Yangpyeong, Gyeonggi‐do	KIOM2012KR4‐17	MH321528	ZS_YP
4		Dongseo, Yuseong, Daejeon	KIOM2012KR5‐17	MH321529	ZS_DJ
5		Gonyang, Sacheon, Gyeongsangnam‐do	KIOM2012KR9‐12	MH321530	ZS_SC
6		Saekdal, Seogwipo, Jeju‐do	KIOM2012KR8‐45	MH321531	ZS_SGP
7	*Zanthoxylum piperitum*	Agyang, Hadong, Gyeongsangnam‐do	KIOM2012KR12‐44	MH321532	ZP_HD
8		Banpo, Gongju, Chungcheongnam‐do	KIOM2012KR13‐35	MH321533	ZP_GJ
9		Songnisan, Boeun, Chungcheongbuk‐do	KIOM2012KR13‐23	MH321534	ZP_BU
10		Anui, Hamyang, Gyeongsangnam‐do	KIOM2012KR6‐14	MH321535	ZP_HY
11		Buk, Ulleung, Gyeongsangbuk‐do	KIOM2013KR5‐47	MH321536	ZP_UL
12		Gachang, Dalseong, Daegu	KIOM2013KR10‐38	MH321537	ZP_DG
13		Ongnyong, Gwangyang, Jeollanam‐do	KIOM2013KR2‐22	MH321538	ZP_GY
14	*Zanthoxylum armatum*	Hangyeong, Jeju, Jeju‐do	MBC_KIOM‐2016‐375	MH321539	ZA_JJ
15		Geunnam, Uljin, Gyeongsangbuk‐do	MBC_KIOM‐2017‐1	MH321540	ZA_UJ
16		Miro, Samcheok, Gangwon‐do	MBC_KIOM‐2017‐2	MH321541	ZA_SC
17		Hai, Goseong, Gyeongsangnam‐do	MBC_KIOM‐2017‐5	MH321542	ZA_GS
18		Gogun, Jindo, Jeollanam‐do	KIOM200701000313	MH321543	ZA_JD
19	*Zanthoxylum bungeanum*	Jiangjin, Chongqing, China	KIOM201201005616	MH321544	ZB_JK
20		Sichuan, China	KIOM2018‐GM	MH321545	ZB_SC#1
21		Sichuan, China	KIOM2018‐WG	MH321546	ZB_SC#2
22		Sichuan, China	KIOM2018‐ON	MH321547	ZB_SC#3

### Genomic DNA extraction from tissue

For the SCAR marker and real‐time PCR assays, genomic DNA of 22 samples was prepared using the DNeasy^®^ Plant Mini Kit (Qiagen, Valencia, CA, USA). Prior to extraction using the kit, approximately 100 mg of sample in 1.6 mL of AP1 buffer was completely crushed using a Precellys™ grinder (Bertin Technologies, Montigny‐le‐Bretonneux, France), as described previously.^26^ Purified genomic DNA was quantitated by measuring absorbance at 260 nm with an ND‐1000 UV‐visible spectrophotometer (NanoDrop, Wilmington, DE, USA). The quality of the genomic DNA was confirmed by 1.5% agarose gel electrophoresis with a Gel Doc system (Syngene, Cambridge, UK). To decrease the rate of error in quantitative analyses, pure genomic DNA with an *A*_260_/*A*_280_ ratio of 1.8–2.0 was used as a template for standard curve analysis in real‐time PCR. All samples were diluted to a final concentration of *ca* 15 ng µL^−1^.

### Genomic DNA extraction from oil

To concentrate Chinese pepper oil, 50 mL of sample was centrifuged at 3000 rpm for 20 min. After centrifugation, the supernatant was discarded and 1 mL of Lysis Buffer L of the Olive Oil DNA Isolation Kit was added to the pellet (Norgen, Thorold, ON, Canada). The pellet was resuspended vigorously using a vortex for 60 s, and incubated at 65 °C for 10 min. To extract genomic DNA, subsequent steps were performed according to the manufacturer's instructions.

### ITS2 sequence analysis

ITS2 was amplified from 22 samples using universal primers ITS2‐S2F (5′‐ATG CGA TAC TTG GTG TGA AT‐3′) and ITS4 (5′‐TCC TCC GCT TAT TGA TAT GC‐3′).^27^ PCR of the ITS2 region was performed in a total reaction volume of 40 µL, containing approximately 15 ng of template, 0.5 µmol L^–1^ ITS2‐S2F and ITS4 primers, and Solg™ 2× Taq PCR Smart‐Mix I (Solgent, Daejeon, Korea). PCR conditions were as follows: initial denaturation at 95 °C for 2 min; followed by 35 cycles of denaturation at 95 °C for 40 s, annealing at 53 °C for 40 s and elongation at 72 °C for 1 min; and a final elongation at 72 °C for 5 min. The amplified DNA fragments were subjected to electrophoresis, and then extracted from the agarose gel using the Gel Extraction Kit (Qiagen). The eluted DNA fragments were ligated into the pGEM^®^‐T Easy Vector (Promega, Madison, WI, USA), and transformed into *Escherichia coli* competent cells using HIT™‐DH5‐α Value 10^8^ (RBC Bioscience, Taipei, Taiwan). The recombinant *E. coli* were incubated for 18 h at 37 °C, and then selected in the presence of 100 µg mL^–1^ ampicillin, 40 µg mL^–1^ X‐gal and 0.5 mmol L^–1^ IPTG on LB agar medium. To confirm the presence of the representative sequence, six white colonies were analyzed per sample. The insert regions of plasmid DNA were sequenced using dideoxynucleotide chain termination sequencing (Sanger sequencing) with standard SP6 and T7 primers. Raw ITS2 sequences were edited using the BioEdit software (version 7.2.5) to trim unnecessary sequences, and then sequences of individual samples were aligned by ClustalW multiple alignment.^28^

### Development of SCAR marker for conventional PCR assay

Species‐specific primers used for the SCAR marker assay were designed based on the variability of ITS2 sequences in four *Zanthoxylum* species. Several candidate primers were selected based on species‐specific substitutions; the number of combinations was 4 in *Z. schinifolium*, 20 in *Z. piperitum*, 4 in *Z. armatum* and 28 in *Z. bungeanum*. To verify primer specificity, conventional PCR was performed in a total reaction volume of 25 µL, containing approximately 15 ng of template, 0.4 µmol L^–1^ of each forward and reverse primer and Solg™ 2× Taq PCR Smart‐Mix I (Solgent). SCAR marker assays were conducted with a Proflex PCR system (Applied Biosystems, Foster City, CA, USA) with the following conditions: initial denaturation at 95 °C for 2 min; 35 cycles of denaturation at 95 °C for 20 s, annealing at 63 °C for 30 s and elongation at 72 °C for 20 s; and final elongation at 72 °C for 5 min. After PCR, products and 100 bp DNA ladder (Solgent) were separated on a 1.5% agarose gel in 0.5× Tris acetate–EDTA buffer as a running buffer.

### Establishment of real‐time PCR assay

Real‐time PCR was performed in a total reaction volume of 25 µL, containing approximately 15 ng of template, 0.4 µmol L^–1^ each of forward and reverse primers and QuantiNova™ SYBR Green PCR Master Mix (Qiagen). To prepare a standard curve, species‐specific DNA fragments subcloned into the pGEM®‐T Easy Vector (Promega) were used as template. Species‐specific DNA fragments of a serially diluted template (10^8^, 10^7^, 10^6^, 10^5^, 10^4^ and 10^3^ copy) were used for quantitation and to confirm the efficiency of equivalent amplification.^29^, ^30^ Real‐time PCR assays were conducted with a Rotor‐Gene Q (Qiagen, 40 724 Hilden, Germany) machine with the following conditions: initial denaturation at 95 °C for 2 min, followed by 40 cycles of denaturation at 95 °C for 10 s and annealing/extension at 60 °C for 10 s, and stepwise melting from 75 to 95 °C with 1 °C increments per 5 s. Thresholds were determined, based on a noticeable difference relative to the no template control (NTC), using Auto‐Find Threshold of the Rotor‐Gene Q Series software (version 2.3.1). Real‐time PCR was carried out in three times with duplicate per running. *R* ^2^ coefficient, slope (*S*) and amplification efficiency were calculated using the standard curve calibrated from the serially diluted template. The limit of quantitation (LOQ) and LOD were respectively obtained from 15 and 60 replicates using species‐specific DNA fragments as templates (LOQ: 80, 50 and 40 copy; LOD: 20, 10, 5 copies and 1 copy), according to the guidelines of Minimum Information for Publication of Quantitative Real‐Time PCR Experiments and the Joint Research Centre (JRC).^29^, ^30^

### Validation of PCR assay and monitoring of adulteration using herbal medicines and Chinese pepper oils

To test the species discrimination abilities of the conventional and real‐time PCR assays, 10 herbal medicines distributed as Zanthoxyli Pericarpium from herbal markets in Korea and China and 12 samples of edible Chinese pepper oil produced at a Korean *Z. schinifolium* farm were purchased. All edible Chinese pepper oil used in this study was extracted by the expeller or oil‐press methods. Validation and monitoring using the conventional and real‐time PCR assays were carried out as described above using the four species‐specific SCAR markers.

## RESULTS AND DISCUSSION

### Analysis of ITS2 sequences and SCAR primer design

Efficiency of PCR amplification is an important factor in the evaluation of DNA barcoding regions.^11^, ^31^ Recent work showed that ITS (or ITS2) can be used for species identification and discrimination between authentic and substitute/adulterant plants.^7^, ^11^ In some plant taxa, however, this approach is of limited usefulness for species identification due to sequence variation in the universal primer regions, as well as the secondary structure of the genome.^8^, ^32^ Despite the fact that ITS (or ITS2) exhibits poor PCR amplification in some plant taxa, it can be used for higher‐resolution species identification than other DNA barcode regions.^32^, ^33^

We confirmed that the 22 samples of four *Zanthoxylum* species could be successfully amplified with the ITS2‐SF and ITS4 universal primers. The trustworthy sequences of individual samples obtained with the T‐vector cloning system have been submitted to the GenBank database; accession numbers are listed in Table 1.^28^ The lengths of the ITS2 regions in *Z. schinifolium*, *Z. piperitum*, *Z. armatum* and *Z. bungeanum* were 385, 383, 388 and 385 bases, respectively. Multiple alignment of these 22 sequences of the ITS2 region from four *Zanthoxylum* species revealed high identity (supporting information, Fig. S1). In previous work, we obtained similar results for *mat*K and *rbc*L (data not shown), but ITS2 exhibited more species‐specific sequence variation than those two loci, and was therefore most suitable for developing SCAR markers for rapid and accurate discrimination of the four *Zanthoxylum* species.

We selected candidate primer regions with species‐specific sequences in the target species using multiple alignment analysis of 22 sequences from four *Zanthoxylum* species (supporting information, Fig. S1). These regions have conserved inter‐species sequences without intra‐species variation. In particular, the specificity of SCAR primers was improved by locating the target‐specific nucleotide substitutions at the 3′‐end, which provides annealing specificity with target template DNA (Table 2; supporting information, Fig. S1).^13^, ^34^ We tested the specificity of the candidate SCAR markers in conventional and real‐time PCR assays using 22 samples from four *Zanthoxylum* species.

**Table 2:** Nucleotide sequences of primers used in the species‐specific SCAR markers and real‐time PCR assay

Scientific name	Primer	Sequence (5′‐3′)	Marker name	Amplicon size (bp)
*Z. schinifolium*	ZS_F1	CGC GGT TGG CCC AAA TTC	ZS#1	110
	ZS_R1	CTG AGT CTC GAA ACG GAG A		
*Z. piperitum*	ZP_F1	GCC TCC CGT GCG CTC TTA	ZP#2	139
	ZP_R1‐1	CAG GGT CCA TGA GTC CGG T		
*Z. armatum*	ZA_F1‐1	GCC CAA AAT CTG AGT CCG C	ZA#3	111
	ZA_R1	GGG GTC CAT GAG TCC CAG		
*Z. bungeanum*	ZB_F2	GTG AAA ACA AAC CTC TCG AGC TA	ZB#21	100
	ZB_R3	GGG TCG CAA TGC GAG CA		

### Development of species‐specific SCAR markers for *Zanthoxylum* species

**Figure 1:**
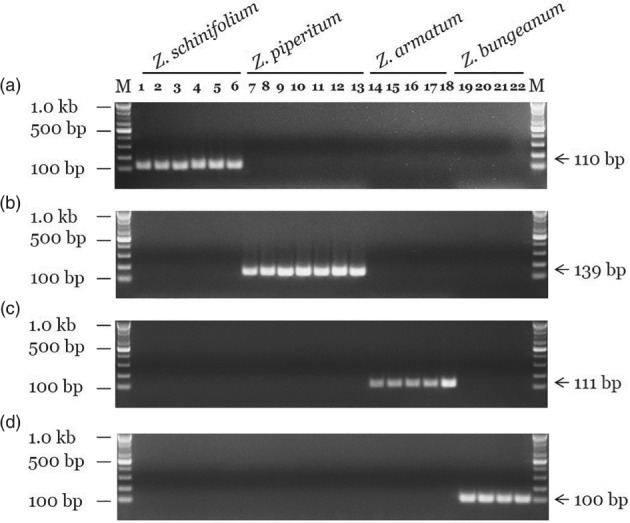
Development and verification of SCAR markers based on species‐specific sequences in the ITS2 region. Lanes 1–6: *Z. schinifolium* samples ZS_GN, HN, YP, DJ, SC and SGP. Lanes 7–13: *Z. piperitum* samples ZP_HD, GJ, BU, HY, UL, DG and GY. Lanes 14–18: *Z. armatum* samples ZA_JJ, UJ, SC, GS and JD. Lanes 19–22: *Z. bungeanum* samples ZB_JK and SC#1–SC#3. Hyphens and arrowheads indicate sizes of DNA ladders and PCR products, respectively. M: 100 bp DNA ladder. (A) *Z. schinifolium*‐specific SCAR markers (ZS#1). (B) *Z. piperitum*‐specific SCAR markers (ZP#2). (C) *Z. armatum*‐specific SCAR markers (ZA#3). (D) *Z. bungeanum*‐specific SCAR markers (ZB#21).

To develop the species‐specific SCAR marker, we screened candidate primers: 4 combinations for *Z. schinifolium*, 20 for *Z. piperitum*, 4 for *Z. armatum* and 28 for *Z. bungeanum* (data not shown). Detailed information about the primers used in this study is provided in Table 2. We performed gradient PCR to search for optimum conditions in the conventional PCR assays. An annealing temperature of 63 °C yielded equivalent band densities between intra‐species samples and were generated target species‐specific amplicon on the gel. Gel images of amplicons obtained in conventional PCR using the SCAR markers for specific detection of the four *Zanthoxylum* species are shown in Fig. 1. Amplified DNA fragments were obtained in the expected sizes only in the target species. For example, an amplicon of 110 bp was obtained with the ZS_F1 and ZS_R1 primers from *Z. schinifolium*, but not from the other three species. In addition, species‐specific amplicons were obtained from *Z. piperitum* (139 bp), *Z. armatum* (111 bp) and *Z. bungeanum* (100 bp).

ITS2 is part of the ITS (consists of ITS1, 5.8S and ITS2), and exists commonly in microorganisms, animals and fungi as well as in plant taxa.^32^, ^33^ Therefore, the PCR product of the SCAR marker‐based ITS2 sequence should detect only the four target *Zanthoxylum* species in herbal medicines or processed foods, including fermentation food and additives. Analysis of the PCR products obtained using the species‐specific SCAR markers revealed that their sequences were identical in each of the *Zanthoxylum* species tested. To test cross‐reactivity with other species or organisms, 26 samples comprising 6 vegetable oil‐related species (*Glycine max*, *Brassica napus*, *Zea mays*, *Vitis vinifera*, *Sesamum indicum* and *Perilla frutescens*), 4 processed food‐related species (*Cordyceps militaris*, *Isaria tenuipes*, *Sanghuangporus sanghuang* and *Sanghuangporus baumii*) and 1 herbal medicine‐related species (*Ophiocordyceps sinensis*) were amplified using four species‐specific SCAR markers (supporting information, Table S1). No amplification was observed on agarose gels, confirming the species specificity of the four SCAR markers (supporting information, Fig. S2). Thus, the species‐specific SCAR markers developed in this study specifically detect the four target *Zanthoxylum* species but not closely related species or other organisms.

### Establishment of real‐time PCR assay

Real‐time PCR can provide quantification without requiring gel electrophoresis, and is also more sensitive and rapid than conventional PCR.^35^ Indeed, previous studies showed that real‐time PCR is 10‐ to 100‐fold more sensitive than conventional PCR.^18^, ^35^ Thus, real‐time PCR is suitable for analysis of food that has undergone intensive processing and contains very small amounts of DNA.^20^, ^36^ We carried out standard curve analyses to quantify real‐time PCR assays using the SCAR markers. In addition, we assessed these markers to determine the optimum temperature (58, 60 and 62 °C) and time (5, 10 and 15 s) for the annealing/extension step, based on the melting temperature and amplicon size (supporting information, Table S2). Also, we optimized the real‐time PCR conditions for primer concentration (0.32, 0.40, 0.48 and 0.56 µmol L^–1^) (supporting information, Table S2). The results revealed that the optimal reaction conditions were as follows: annealing/extension temperature of 60 °C, time of 10 s and 0.4 µmol L^–1^ of each primer.

**Figure 2:**
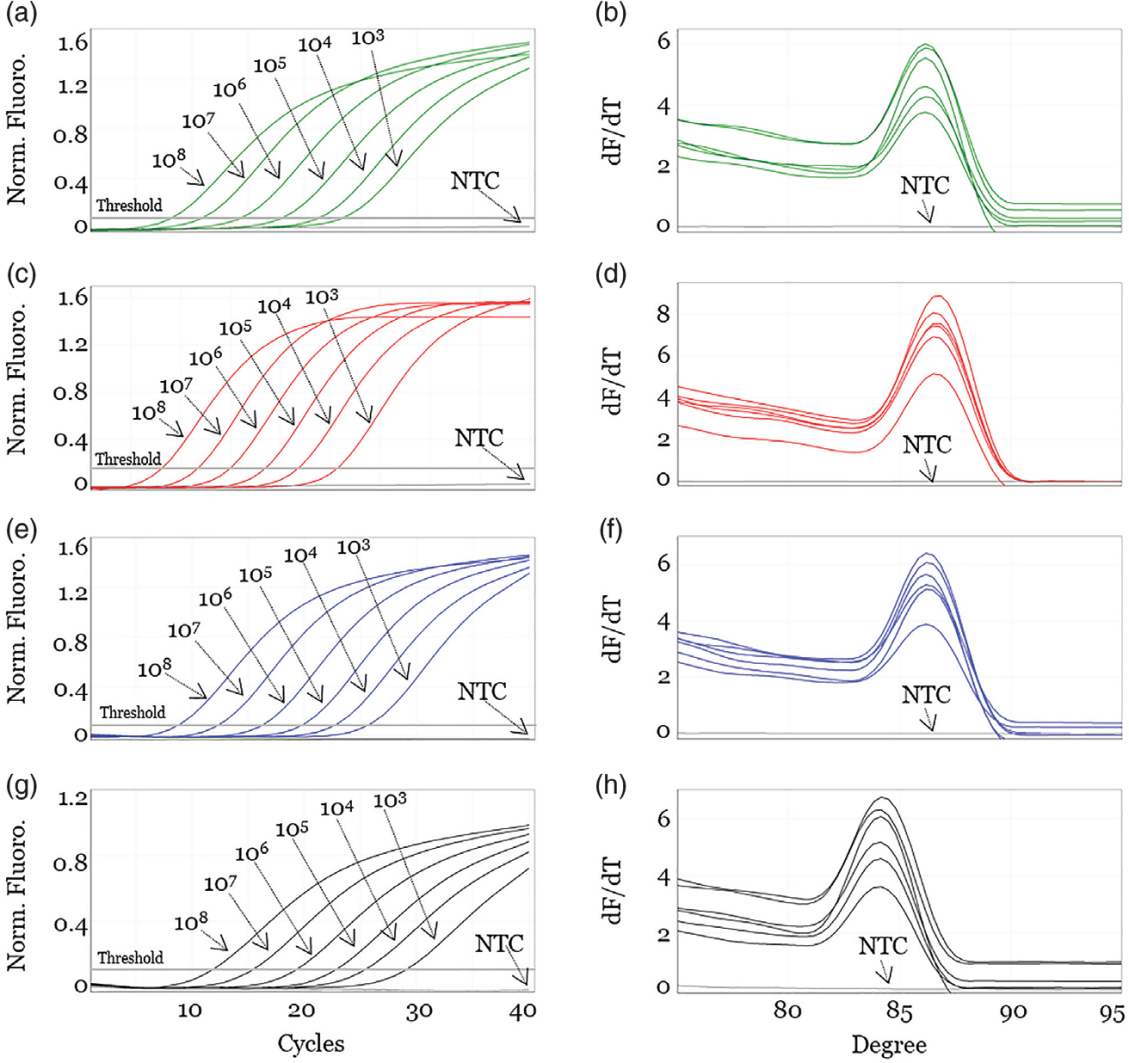
Validation of SCAR markers and monitoring of commercial Chinese pepper oil and Zanthoxyli Pericarpium herbal medicines using the species‐specific SCAR markers developed in this study. Lanes C1–8: control plant samples: *Z. schinifolium* ZS_SC and SGP, *Z. piperitum* ZP_BU and GY, *Z. armatum* ZA_UJ and GS and *Z. bungeanum* ZB_JK and SC#2. Lanes 1–4: Chinese pepper oil extracted by expeller method from seed. Lanes 5–8: Chinese pepper oil extracted by press method from seed containing 10% pericarp. Lanes 9–12: Chinese pepper oil extracted by press method from seed. Lanes 13–22: commercial herbal medicines distributed as Zanthoxyli Pericarpium in Korea and China. Hyphens and arrowheads indicate sizes of DNA ladder and PCR products, respectively. Asterisk (*) represents an inauthentic, and mixed herbal medicine. PCR amplification of SCAR markers specific for (A) *Z. schinifolium*, (B) *Z. piperitum*, (C) *Z. armatum* and (D) *Z. bungeanum*.

The standard curve was generated by plotting the Ct values of serial 10‐fold dilutions against the measured genomic DNA concentrations.^35^, ^37^ Also, linearity of slope (*S*), efficiency (*E*) and *R* ^2^ were calculated from the standard curve (Table 3). For reliable quantification, the ideal values of slope obtained with 10‐fold dilutions should approach −3.3.^29^, ^30^ We obtained −3.28 in *Z. schinifolium*, −3.35 in *Z. piperitum*, −3.34 in *Z. armatum* and −3.35 in *Z. bungeanum*. Efficiency, calculated with the formula *E* = [10^−1/*S*^ − 1] × 100, was 101.3% in *Z. schinifolium*, 98.6% in *Z. piperitum*, 99.0% in *Z. armatum* and 98.6% in *Z. bungeanum*. The correlation coefficient (*R* ^2^) between Ct values and template concentration, which should be greater than 0.98 in a reliable method, was 0.99629 in *Z. schinifolium*, 0.99908 in *Z. piperitum*, 0.99485 in *Z. armatum* and 0.99652 in *Z. bungeanum*. Thus, for all four *Zanthoxylum* species, real‐time PCR was highly sensitive and linear in concentration ranges from 10^8^ to 10^3^ copies (Fig. 2).^29^, ^30^ Also, the specificities of these amplicons were confirmed by melting curve analysis, which revealed peaks at 86.3 °C in *Z. schinifolium*, 87.3 °C in *Z. piperitum*, 86.2 °C in *Z. armatum* and 84.2 °C in *Z. bungeanum*. These results indicate the formation of a single amplicon without nonspecific amplicon or primer‐dimer formation. To determine the LOD and LOQ of analytical sensitivity, each species‐specific SCAR marker was tested in a real‐time PCR assay using the lowest amount of serially diluted template (supporting information, Table S3).^29^, ^30^ The LOQ was 50 copies, corresponding to a relative repeatability standard deviation (RSDr) of below 25%, satisfying the RSDr and lowest copy number (50) of the JRC guidelines.^30^ The LOD was 20 copies, satisfying the lowest copy number (25) and level of confidence of 95%.^30^ Thus, the more cost‐effective SYBR Green‐based approach used in this real‐time PCR assay was capable of sensitive detection of small quantities of DNA.

**Table 3:** Statistics of real‐time PCR assays

Marker name	Slope	Efficiency (%)	*R* ^2^
ZS#1	3.28 ± 0.03	101.3 ± 1.5	0.99629 ± 0.00065
ZP#2	3.35 ± 0.03	98.6 ± 1.1	0.99908 ± 0.00045
ZA#3	3.34 ± 0.01	99.0 ± 1.0	0.99485 ± 0.00516
ZB#21	3.35 ± 0.10	98.6 ± 4.1	0.99652 ± 0.00222

Mean values ± standard deviation (*n* = 6).

Also, we confirmed the specificity of the real‐time PCR assay by comparing amplification of target and non‐target *Zanthoxylum* species (supporting information, Fig. S3). Using primer pair ZS‐F1/‐R1, designed for detection of *Z. schinifolium*, the fluorescence signals generated by amplification were obtained as expected from *Z. schinifolium*, but in the other three *Zanthoxylum* species and NTC, the signals were below the LOD value for *Z. schinifolium*. Also, in each assay, the other three *Zanthoxylum* species, *Z. piperitum*, *Z. armatum* and *Z. bungeanum*, were tested for cross‐amplification using the SCAR markers, and only species‐specific fluorescence signals were obtained (supporting information, Fig. S3).

### Validation of discrimination and monitoring of adulteration using commercial Chinese pepper oil and herbal medicines

Due to high demand for Korean food and medicine, *Z. schinifolium* is sold at a higher price than other *Zanthoxylum* species. In addition, confusion arises for several reasons, such as the difference in the species definition between the Korean and Chinese pharmacopoeias, the homonymous nomenclature for *Z. schinifolium* and Zanthoxyli Pericarpium,and the similarity of morphological characteristics between *Z. armatum* (adulterant) and *Z. bungeanum* (authentic).^25^ Therefore, adulterants or substitutes may be used, either intentionally or accidentally, in processed foods or herbal medicines. We successfully developed species‐specific SCAR markers for conventional PCR and real‐time PCR assays capable of genetically discriminating four *Zanthoxylum* species. In addition, these primers produced amplicons of small size that are easily detected even when DNA has been highly fragmented during processing.

Next, we tested the ability of the species‐specific SCAR markers to genetically discriminate DNA extracted from edible oil. Edible oil extraction methods include expeller and oil press. Given the pharmacological effect of Zanthoxyli Pericarpium, some Chinese pepper oil is manufactured from seed containing the pericarp.^38^ We extracted Chinese pepper oil from well‐ripened seed in two ways, with or without pericarp, using fruit purchased from the *Z. schinifolium* farm, and then tested genetic discrimination using the conventional PCR and real‐time PCR assays.

**Figure 3:**
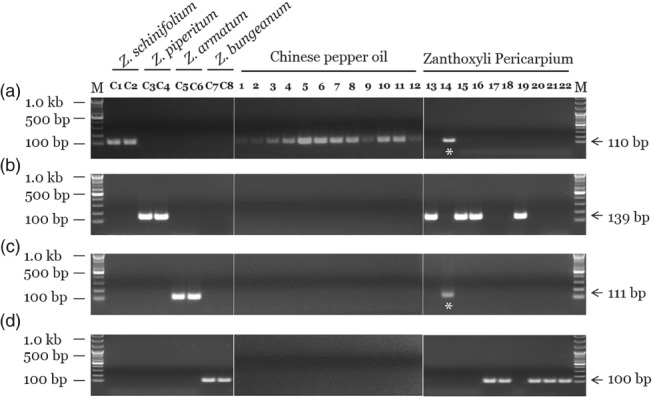
Sensitivity and efficiency analysis of real‐time PCR assay using serially diluted standard DNA and four species‐specific SCAR markers. PCR cycling (A, C, E, G) and melting curves (B, D, F, H) of SCAR markers specific for *Z. schinifolium* (ZS#1; A, B), using primers ZS_F1 and ZS_R1; *Z. piperitum* (ZP#2; C, D), using primers ZP_F1 and ZP_R1‐1; *Z. armatum* (ZA#3; E, F), using ZA_F1‐1 and ZA_R1; and *Z. bungeanum* (ZB#21; G, H), using primers ZB_F2 and ZB_R3. Arrows indicate differential amplification of serially diluted standard DNA. Threshold was determined using Auto‐Find Threshold of the Rotor‐Gene Q software (version 2.3.1).

In the conventional assay, all 12 samples were positive only for the ZS marker, which indicates the presence of genomic DNA from *Z. schinifolium* (Fig. 3, Table 4), demonstrating that the SCAR markers can clearly discriminate even in edible oil. Also, we monitored commercial herbal medicine containing Zanthoxyli Pericarpium from a Korean herbal market (Fig. 3, Table 4). As shown in Fig. 3, all three samples (monitoring samples nos. 13, 16 and 19) produced in Korea were identified as *Z. piperitum*, whereas almost all of the commercial herbal medicines imported from China (nos. 17, 18, 20, 21 and 22) were identified as *Z. bungeanum*. Although it is not included in Chinese pharmacopoeias, *Z. piperitum* was identified in one sample (no. 15). Also, one sample of Zanthoxyli Pericarpium (no. 14) from a Chinese herbal market was identified as *Z. schinifolium* mixed with *Z. armatum*. Thus, we confirmed that adulterants of Zanthoxyli Pericarpium were distributed in Korean herbal markets. To compare the analytic reproducibility of the real‐time PCR assay using the SCAR marker with the conventional PCR assay, we carried out species discrimination assays using the 12 edible oil samples and 10 commercial herbal medicines. The real‐time PCR results for all samples were consistent with the conventional PCR results (Table 4).

**Table 4:** Samples and results from commercial Chinese pepper oil and herbal medicines used to validate conventional and real‐time PCR

No.	Sample type	Production country	Product form	Species identification result	
				Real‐time PCR (quantification; copy number)	Conventional PCR
1	Chinese pepper oil	Korea	Expeller	*Z. schinifolium* (343 ± 45)	*Z. schinifolium*
2		Korea	Expeller	*Z. schinifolium* (265 ± 58)	*Z. schinifolium*
3		Korea	Expeller	*Z. schinifolium* (1626 ± 230)	*Z. schinifolium*
4		Korea	Expeller	*Z. schinifolium* (7538 ± 351)	*Z. schinifolium*
5		Korea	Oil press + 10% pericarp	*Z. schinifolium* (3 372 712 ± 309 174)	*Z. schinifolium*
6		Korea	Oil press + 10% pericarp	*Z. schinifolium* (45 663 ± 6810)	*Z. schinifolium*
7		Korea	Oil press + 10% pericarp	*Z. schinifolium* (29 508 ± 2454)	*Z. schinifolium*
8		Korea	Oil press + 10% pericarp	*Z. schinifolium* (39 185 ± 5944)	*Z. schinifolium*
9		Korea	Oil press	*Z. schinifolium* (1021 ± 89)	*Z. schinifolium*
10		Korea	Oil press	*Z. schinifolium* (6608 ± 882)	*Z. schinifolium*
11		Korea	Oil press	*Z. schinifolium* (3942 ± 282)	*Z. schinifolium*
12		Korea	Oil press	*Z. schinifolium* (725 ± 118)	*Z. schinifolium*
13	Zanthoxyli Pericarpium	Korea	Dried pericarp	*Z. piperitum* (193 509 ± 32 244)	*Z. piperitum*
14		China	Dried pericarp	*Z. schinifolium* (5 101 740 ± 565 195) *+ Z. armatum* (9368 ± 358)†	*Z. schinifolium + Z. armatum* †
15		China	Dried pericarp	*Z. piperitum* (4 874 231 ± 1 410 618)	*Z. piperitum*
16		Korea	Dried pericarp	*Z. piperitum* (1 003 946 ± 159 352)	*Z. piperitum*
17		China	Dried pericarp	*Z. bungeanum* (225 669 ± 19 308)	*Z. bungeanum*
18		China	Dried pericarp	*Z. bungeanum* (147 306 ± 12 658)	*Z. bungeanum*
19		Korea	Dried pericarp	*Z. piperitum* (120 678 ± 8006)	*Z. piperitum*
20		China	Dried pericarp	*Z. bungeanum* (195 928 ± 6184)	*Z. bungeanum*
21		China	Dried pericarp	*Z. bungeanum* (475 764 ± 26 865)	*Z. bungeanum*
22		China	Dried pericarp	*Z. bungeanum* (248 898 ± 12 681)	*Z. bungeanum*

† Inauthentic and mixed herbal medicine.

Mean values ± standard deviation (*n* = 3).

Therefore, the SCAR markers were suitable for both conventional PCR and real‐time PCR assay, and could be used as tools for rapid and accurate species discrimination in processed food and dried herbal medicines. These markers could make a major contribution to standardization of food and herbal medicine.

## CONCLUSIONS

We analyzed the ITS2 sequence of four *Zanthoxylum* species, *Z. schinifolium* (Chinese pepper), *Z. piperitum*, *Z. armatum* and *Z. bungeanum* (Sichuan pepper), which are used in processed food and herbal medicines. We then designed SCAR markers based on the species‐specific sequence substitutions in the ITS2 locus. The SCAR markers were designed to generate amplicons shorter than 200 bp, which are advantageous for genetic discrimination of damaged DNA present in processed food and herbal medicines. The SCAR markers were suitable for both conventional and real‐time PCR assays, and had high detection sensitivity (at the picogram level). In addition, we validated the SCAR markers by analyzing commercial products, including 12 edible Chinese pepper oils and 10 commercial herbal medicines. The results revealed that some commercial herbal medicines are mixtures of two or more pericarps of *Zanthoxylum* species, and that Zanthoxyli Pericarpium is contaminated with inauthentic adulterants such as *Z. armatum*. Thus, the SCAR marker‐based PCR assays developed in this study provide useful genetic tools for quality control and standardization of food and herbal medicines.

## Supporting information


**Figure S1.** Multiple alignment of ITS2 sequences and positions of SCAR primers. Boxes indicate primer sequences and orientations.

**Figure S2.** Verification of the specificity of the SCAR markers in conventional PCR assays, using 26 samples of oil related plant species and other organisms. Lanes C1–8: Control plant samples: *Z. schinifolium* ZS_SC and SGP, *Z. piperitum* ZP_BU and GY, *Z. armatum* ZA_UJ and GS, and *Z. bungeanum* ZB_JK and SC#2. Lanes 1–27: plant species used as vegetable oil: G. max (soybean), B. napus (rape), Z. mays (corn), V. vinifera (Grape), S. indicum (sesame), P. frutescens (perilla). Lanes 18–26: fungus species used as food supplements or herbal medicines: C. militaris (Militaris *Dong Chung Ha Cho*), *I. tenuipes* (*Dong Chung Ha Cho*), O. sinensis (Cordyceps), *S. sanghuang* (*Sang Hwang Beo Seot*), *S. baumii* (*Jang Su Jin Heut Beo Seot*). (A–D) PCR amplification of SCAR markers specific for (A) *Z. schinifolium* (B) *Z. piperitum*, (C) *Z. armatum*, and (D) *Z. bungeanum*.

**Figure S3.** Verification of the specificity of SCAR markers in real‐time PCR, using 22 samples of four *Zanthoxylum* species. (A–D) PCR cycling SCAR markers specific for (A) *Z. schinifolium*, (B) *Z. piperitum*, (C) *Z. armatum*, and (D) *Z. bungeanum*. Arrows indicate PCR amplification of the species‐specific SCAR marker in the target species and the other three *Zanthoxylum* species. Threshold was determined manually using Rotor‐Gene Q software (version 2.3.1), based on standard curve analysis.

**Table S1.** List of oil related plant species and other organisms analyzed in this study to validate the specificity of the SCAR markers.
**Table S2.** Optimization according to primer concentration, and temperature or time for annealing‐extension.
**Table S3.** Estimation of LOQ and LOD of the real‐time PCR assay.
