# Supplementary figures and images for: Development of conventional PCR and real‐time PCR assays to discriminate the origins of Chinese pepper oil and herbal materials from Zanthoxylum
Source: J Sci Food Agric. 2018 Dec 13;99(4):2021–9. doi: 10.1002/jsfa.9458 (PMC6590328; doi:10.1002/jsfa.9458)

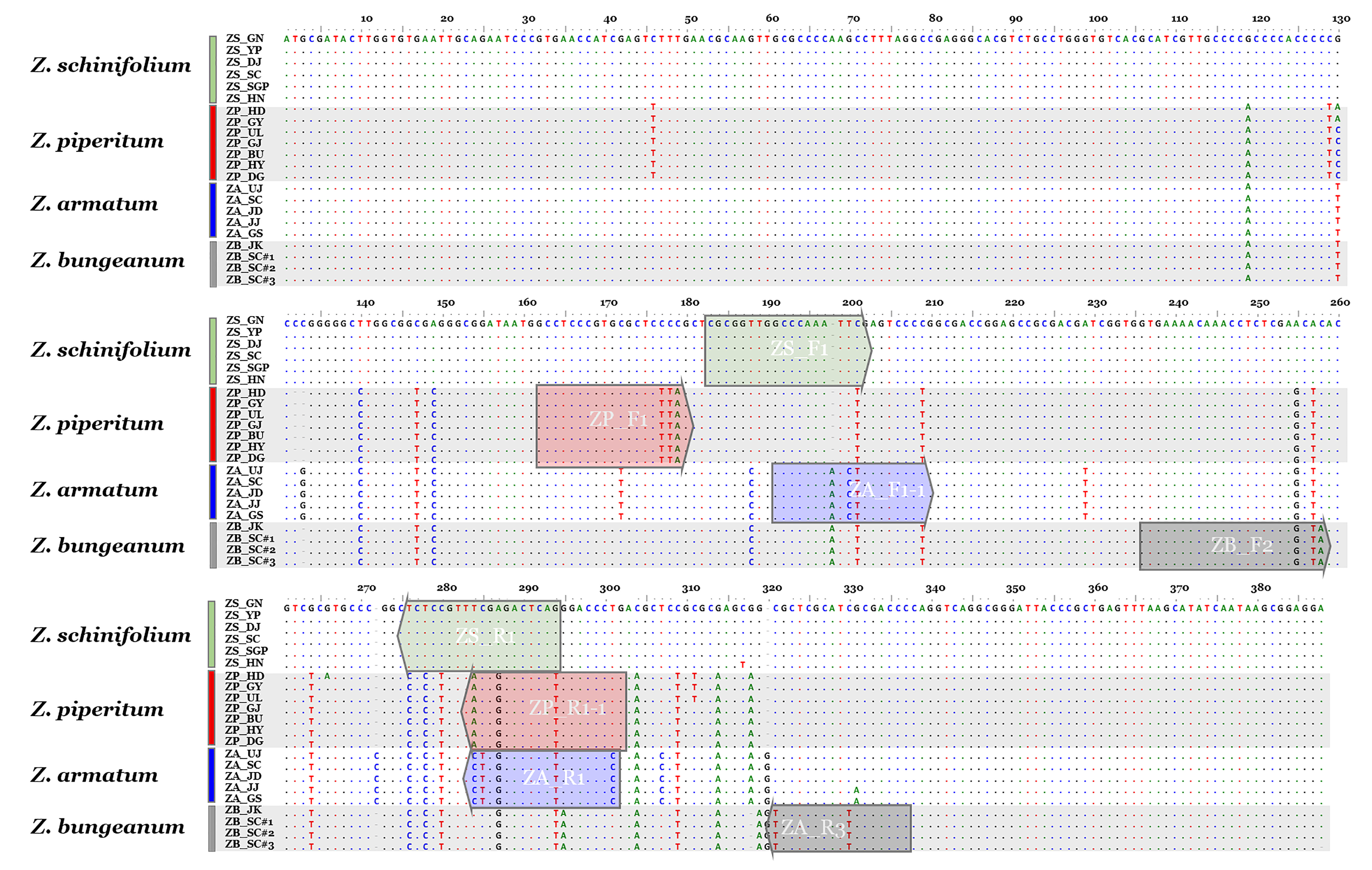

Supplement: Supplementary file 1 — Figure S1. Multiple alignment of ITS2 sequences and positions of SCAR primers. Boxes indicate primer sequences and orientations. [file JSFA-99-2021-s003.jpg]

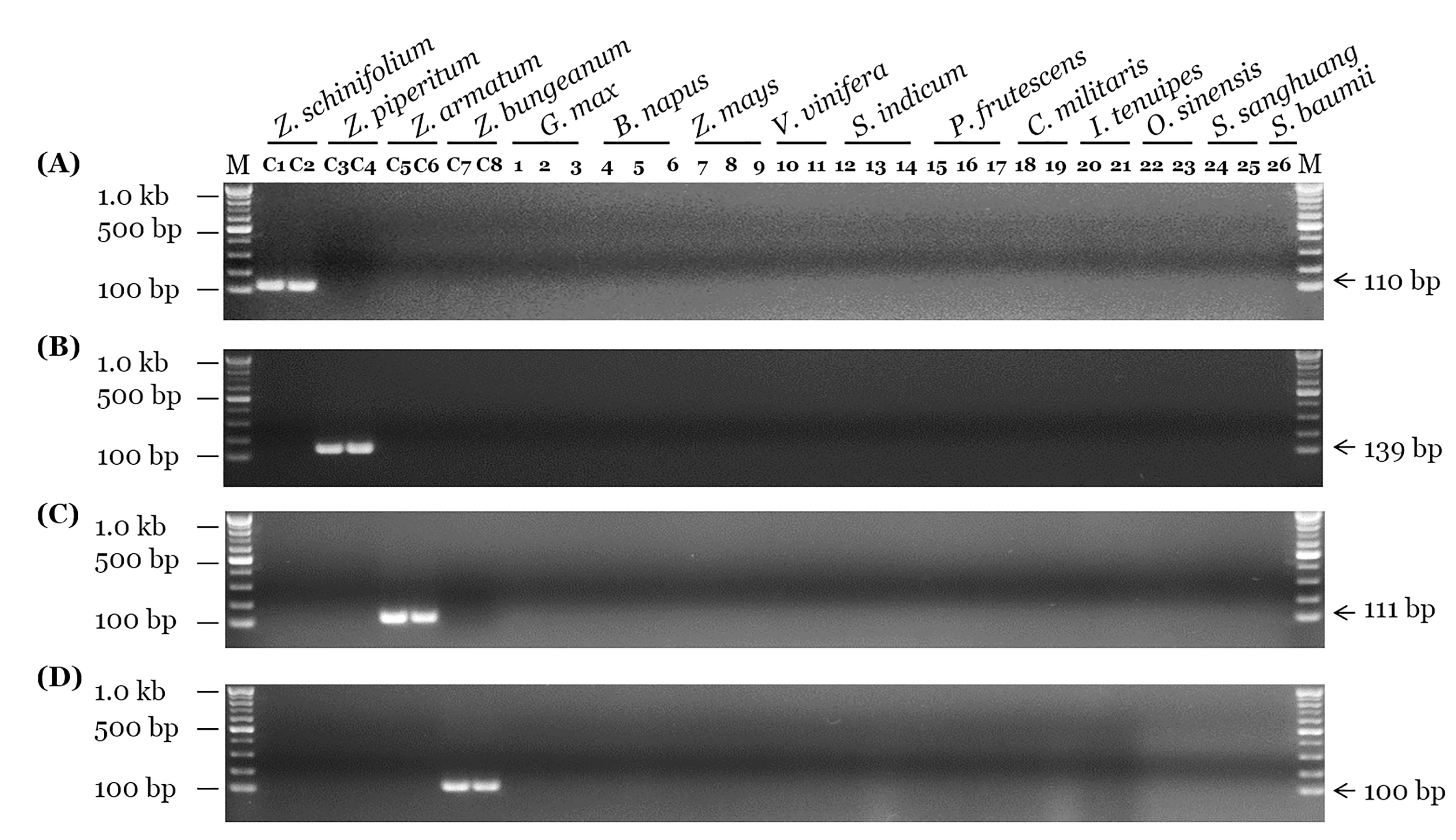

Supplement: Supplementary file 2 — Figure S2. Verification of the specificity of the SCAR markers in conventional PCR assays, using 26 samples of oil related plant species and other organisms. Lanes C1–8: Control plant samples: Z. schinifolium ZS_SC and SGP, Z. piperitum ZP_BU and GY, Z. armatum ZA_UJ and GS, and Z. bungeanum ZB_JK and SC#2. Lanes 1–27: plant species used as vegetable oil: G. max (soybean), B. napus (rape), Z. mays (corn), V. vinifera (Grape), S. indicum (sesame), P. frutescens (perilla). Lanes 18–26: fungus species used as food supplements or herbal medicines: C. militaris (Militaris Dong Chung Ha Cho), I. tenuipes (Dong Chung Ha Cho), O. sinensis (Cordyceps), S. sanghuang (Sang Hwang Beo Seot), S. baumii (Jang Su Jin Heut Beo Seot). (A–D) PCR amplification of SCAR markers specific for (A) Z. schinifolium (B) Z. piperitum, (C) Z. armatum, and (D) Z. bungeanum. [file JSFA-99-2021-s001.jpg]

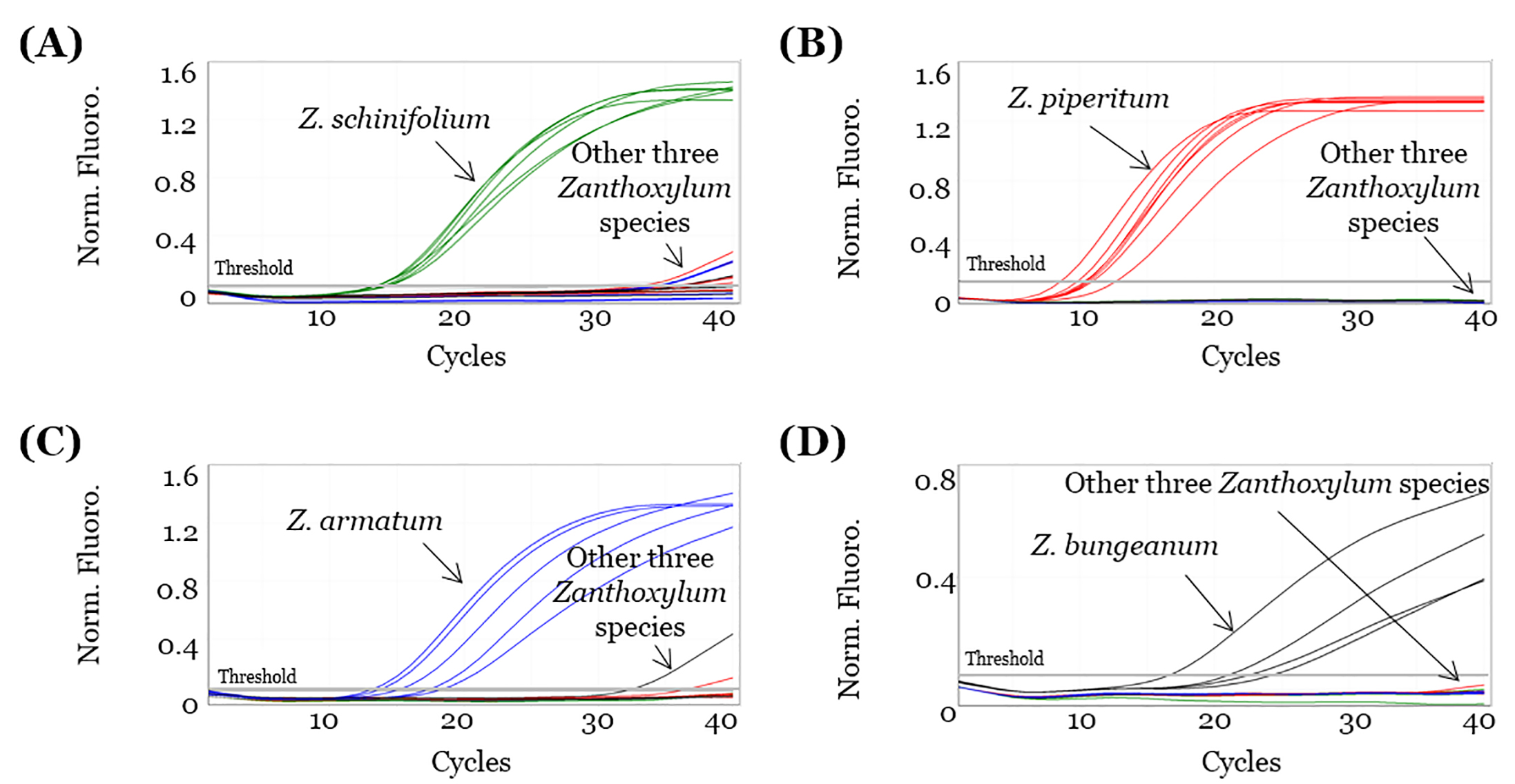

Supplement: Supplementary file 3 — Figure S3. Verification of the specificity of SCAR markers in real‐time PCR, using 22 samples of four Zanthoxylum species. (A–D) PCR cycling SCAR markers specific for (A) Z. schinifolium, (B) Z. piperitum, (C) Z. armatum, and (D) Z. bungeanum. Arrows indicate PCR amplification of the species‐specific SCAR marker in the target species and the other three Zanthoxylum species. Threshold was determined manually using Rotor‐Gene Q software (version 2.3.1), based on standard curve analysis. [file JSFA-99-2021-s004.jpg]
