# Supplementary material for: Development of conventional PCR and real‐time PCR assays to discriminate the origins of Chinese pepper oil and herbal materials from Zanthoxylum
Source: J Sci Food Agric. 2018 Dec 13;99(4):2021–9. doi: 10.1002/jsfa.9458 (PMC6590328; doi:10.1002/jsfa.9458)
Supplement: Supplementary file 4 — Table S1. List of oil related plant species and other organisms analyzed in this study to validate the specificity of the SCAR markers. Table S2. Optimization according to primer concentration, and temperature or time for annealing‐extension. Table S3. Estimation of LOQ and LOD of the real‐time PCR assay. [file JSFA-99-2021-s002.docx]

**Supplementary Table 1.** List of oil related plant species and other organisms analyzed in this study to validate the specificity of the SCAR markers.

| Organism | Scientific name | Common name | Voucher number | Lane |
| --- | --- | --- | --- | --- |
| Plant | *Glycine max* (L.) Merr. | Soybean | KIOM201601018488 | 1 |
|  |  |  | KIOM201601018489 | 2 |
|  |  |  | KIOM201601018490 | 3 |
|  | *Brassica napus* L. | Rape | KIOM201701019397 | 4 |
|  |  |  | KIOM201701019398 | 5 |
|  |  |  | KIOM201801020733 | 6 |
|  | *Zea mays* L. | Maize | KIOM201701018944 | 7 |
|  |  |  | KIOM201701018945 | 8 |
|  |  |  | KWJ_KIOM_2018-1 | 9 |
|  | *Vitis vinifera* L. | Grape | KWJ_KIOM_2018-2 | 10 |
|  |  |  | KWJ_KIOM_2018-3 | 11 |
|  | *Sesamum indicum* L. | Sesame | KIOM201701018928 | 12 |
|  |  |  | KIOM201701018839 | 13 |
|  |  |  | KIOM201701018840 | 14 |
|  | *Perilla frutescens* (L.) Britton | Perilla | KIOM201201005144 | 15 |
|  |  |  | KIOM201201005145 | 16 |
|  |  |  | KIOM201201005146 | 17 |
| Fungus | *Cordyceps militaris* (L.) Fr. | - | KACC43319 | 18 |
|  |  |  | KACC44463 | 19 |
|  | *Isaria tenuipes* Peck | - | KACC43336 | 20 |
|  |  |  | KACC44476 | 21 |
|  | *Ophiocordyceps sinensis* (Berk.) G.H. Sung, J.M. Sung, Hywel-Jones and Spatafora | Cordyceps | 2-2016-F027 | 22 |
|  |  |  | 2-2016-F028 | 23 |
|  | *Sanghuangporus sanghuang* (Sheng H. Wu, T. Hatt. & Y.C. Dai) Sheng H. Wu, L.W. Zhou & Y.C. Dai | - | ASI26025 | 24 |
|  |  |  | KCTC6719 | 25 |
|  | *Sanghuangporus baumii* (Pilát) L.W. Zhou & Y.C. Dai | - | ASI26087 | 26 |

**Supplementary Table 2.** Optimization according to primer concentration, and temperature or time for annealing-extension.

|  |  | ZS#1 | | ZP#2 | | ZA#3 | | ZB#21 | |
| --- | --- | --- | --- | --- | --- | --- | --- | --- | --- |
|  |  | Efficiency (%) | R^2^ | Efficiency (%) | R^2^ | Efficiency (%) | R^2^ | Efficiency (%) | R^2^ |
| Temperature for annealing-extension | 58°C | 116 | 0.99286 | 119 | 0.98997 | 106 | 0.99882 | 94 | 0.99813 |
|  | 60°C | 101 | 0.99585 | 98 | 0.99953 | 99 | 0.99815 | 100 | 0.99414 |
|  | 62°C | 88 | 0.99089 | 105 | 0.99816 | 102 | 0.99777 | 95 | 0.99648 |
| Time for annealing-extension | 5 sec | 93 | 0.99762 | 94 | 0.99634 | 102 | 0.99688 | 95 | 0.99828 |
|  | 10 sec | 101 | 0.99585 | 98 | 0.99953 | 99 | 0.99815 | 100 | 0.99414 |
|  | 15 sec | 99 | 0.99039 | 93 | 0.99346 | 99 | 0.99582 | 105 | 0.99793 |
| Primer concentration | 0.32 uM | 97 | 0.99606 | 106 | 0.99594 | 97 | 0.99809 | 93 | 0.99841 |
|  | 0.40 uM | 101 | 0.99585 | 98 | 0.99953 | 99 | 0.99815 | 100 | 0.99414 |
|  | 0.48 uM | 109 | 0.99385 | 94 | 0.99465 | 109 | 0.99828 | 106 | 0.99846 |
|  | 0.56 uM | 109 | 0.99680 | 94 | 0.99762 | 101 | 0.99633 | 105 | 0.99803 |

Mean values (n=2).

**Supplementary Table 3.** Estimation of LOQ and LOD of the real-time PCR assay.

| Marker name | Copy number | Positive signal /replicated test | Ratio of positive signal (%) | Ct (mean ± SD) | RSDr |
| --- | --- | --- | --- | --- | --- |
| ZS#1 | 80 | 15/15 | 100 | 29.50 ± 0.32 | 1.08 |
|  | 50 | 15/15 | 100 | 30.90 ± 0.34 | 1.10 |
|  | 40 | 15/15 | 100 | 31.14 ± 0.44 | 1.41 |
|  | 20 | 60/60 | 100 | 31.87 ± 1.03 | 3.23 |
|  | 10 | 56/60 | 93 | - | - |
|  | 5 | 51/60 | 85 | - | - |
|  | 1 | 48/60 | 80 | - | - |
| ZP#2 | 80 | 15/15 | 100 | 29.29 ± 0.44 | 1.49 |
|  | 50 | 15/15 | 100 | 29.73 ± 1.69 | 5.68 |
|  | 40 | 15/15 | 100 | 30.35 ± 1.64 | 5.40 |
|  | 20 | 60/60 | 100 | 31.10 ± 0.80 | 2.57 |
|  | 10 | 52/60 | 87 | - | - |
|  | 5 | 47/60 | 78 | - | - |
|  | 1 | 33/60 | 55 | - | - |
| ZA#3 | 80 | 15/15 | 100 | 29.66 ± 0.33 | 1.11 |
|  | 50 | 15/15 | 100 | 30.59 ± 0.81 | 2.64 |
|  | 40 | 15/15 | 100 | 31.02 ± 0.59 | 1.90 |
|  | 20 | 60/60 | 100 | 31.34 ± 0.47 | 1.49 |
|  | 10 | 56/60 | 93 | - | - |
|  | 5 | 46/60 | 76 | - | - |
|  | 1 | 30/60 | 50 | - | - |
| ZB#21 | 80 | 15/15 | 100 | 32.13 ± 0.23 | 0.99 |
|  | 50 | 15/15 | 100 | 32.31 ± 0.35 | 1.08 |
|  | 40 | 15/15 | 100 | 32.44 ± 0.41 | 1.26 |
|  | 20 | 60/60 | 100 | 33.10 ± 0.89 | 2.68 |
|  | 10 | 54/60 | 90 | - | - |
|  | 5 | 54/60 | 90 | - | - |
|  | 1 | 11/60 | 18 | - | - |
